# Farmers’ suicides in the Vidarbha region of Maharashtra, India: a qualitative exploration of their causes

**DOI:** 10.5249/jivr.v4i1.68

**Published:** 2012-01

**Authors:** Amol R. Dongre, Pradeep R. Deshmukh

**Affiliations:** ^*a*^Department of Community Medicine, Sri Manakula Vinayagar Medical College, Pondicherry, India.; ^*b*^Dr Sushila Nayar School of Public Health, Mahatma Gandhi Institute of Medical Sciences, Sewagram, India.

## Abstract

**Background:**

To explore the various perceived reasons for farmers’ suicides in the Vidarbha region of Maharashtra, their common factors, and to suggest solutions.

**Methods:**

The present formative research was undertaken in the 23 villages surrounding the Anji Primary Health Centre, located in the Vidarbha region of Maharashtra, India. A triangulation of free list and pile sort exercises was used. The data was analyzed by Anthropac 4.98.1/X software. This was followed by a semi-structured focus group discussion. To increase the validity of the results, these findings were presented to the participants and later they were circulated to the 26 farmers’ clubs in the villages for comment and discussion during their monthly, village based meetings.

**Results:**

Farmers perceived debt, addiction, environmental problems, poor prices for farm produce, stress and family responsibilities, government apathy, poor irrigation, increased cost of cultivation, private money lenders, use of chemical fertilizers and crop failure as the reasons for farmers’ suicides. Participants suggested solutions such as self-reliance and capacity building among farmers, a monitoring and support system for vulnerable farmers, support and counseling services, a village-level, transparent system for the disbursement of relief packages.

**Conclusions:**

Farmers’ suicides in Vidarbha are caused by the complex interplay of social, political and environmental constraints. Hence, a comprehensive intervention to ensure self reliance and capacity building among farmers in modern farming techniques , monitoring and support system for vulnerable farmers, a village-level, transparent system for disbursement of relief packages is required to prevent farmer suicides in the near future. Apart from this, there is a need to strengthen the National Mental Health Program at primary health care level to offer support and counseling to vulnerable farmers in rural area.

## Introduction

In India, farmer suicides had been reported from various states, viz. Andhra Pradesh, Punjab Karnataka and Orissa.^1^Maharashtra, one of India’s most prosperous states is currently facing an epidemic of farmer suicides especially in the Vidarbha region. Studies in India, Sri Lanka, Canada, England and Australia have identified farming as one of the most high-risk industries when it comes to having a suicide rate higher than in the general population.^2^In India, national data show that the suicide rate was 9.7/100000 population per year in 1995. The population of the Vidarbha region is 1 200 000, so the expected number of suicides was 116 per year, but it was found to be 572 in 2005, 1065 in 2006 and 600 in year 2007.^3^A report by the Tata Institute of Social Sciences, Mumbai identified the reasons for farmer’s suicides: repeated crop failures, inability to meet the rising cost of cultivation, and debt.^4^Even when the government announced relief packages for the affected families and remedial measures, this did not lead to any immediate positive effect on suicide behavior. It was reported that farmers’ concerns were not taken into account while designing these relief packages.^5^

The Kasturba Rural Health Training Centre (KRHTC) in Anji successfully implemented the ‘Community Led Initiatives for Child Survival’ (CLICS) program in the 23 villages surrounding the Anji Primary Health Centre in the Wardha district during the period 2003 to 2008. As a part of community mobilization, Kisan Vikas Manch (KVM, farmers development forums) were formed in each village to ensure the involvement of men in the program. This community-based platform was used for Participatory Research and Action, health message dissemination and village-based agricultural guidance.^6^As a step towards self reliance and sustainability, KVMs under KRHTC were up-graded from informal groups to self-help groups. KVMs guaranteed savings and ensured loans on flexible conditions to its poor members and subsequently could prevent attempted suicides by two indebted farmers. In September 2008, we invited KVM members from the 23 surrounding villages to participate in a discussion to explore the various perceived reasons for farmers’ suicides in the Vidarbha region of Maharashtra, their mutual relationships and to suggest solutions.

## Methods

The present formative research was undertaken in the 23 surrounding villages of KRHTC, Anji, which is located in the Wardha district of the Vidarbha region, about 758 km east from the state capital, Mumbai. This economically backward region is located in the north eastern part of Maharashatra state and its economy depends on agriculture.^7^Wardha is a sister city for Sevagram, and both were used as major centers for the Indian Independence Movement, especially as headquarters for an annual meeting of the Indian National Congress in 1934, and Mahatma Gandhi’s Ashram. In recent time, repeated crop failures, the rising cost of cultivation and debt have created a situation that is leading to farmers committing suicide in the Vidarbha region.

There were 26 KVMs in 23 villages. Each KVM had 15-20 small scale male farmers as members. Out of this, 17 KVMs had been active in organizing village-level technical agricultural guidance for all other farmers. We invited in one member from each of these 17 KVMs, who was willing to participate and talk freely on this issue. A one day meeting was organized at KRHTC, Anji on a day and at a time convenient to the participants. Representatives from ten KVMs attended the meeting. This sample size was adequate for pile sorting as after fifth participants, results were likely to get repeated with more than 0.75 correlations of results.^8^After obtaining written consent, a triangulation of free list and pile sorting exercises was used to identify various perceived reasons for farmers’ suicides, their perceived relationships to these factors and possible solutions to address these problems.^9^In the free list exercise, the participants were asked to make an individual free list of the various reasons for farmers’ suicides. Eleven reasons with relatively high Smith’s S value were then pile sorted. Smith’s S (Smith’s saliency score) refers to the importance, representativeness or prominence of items to individuals or to the group, and is measured in three ways: word frequency across lists, word rank within lists and a combination of these two.^10^In the pile sorting exercise, the individual participants were asked to group those selected reasons which they felt went together and suggest the solutions to prevent them. It was followed by a semi-structured focus group discussion (FGD) with these ten members. A note taker recorded the discussion. The data was analyzed by Anthropac 4.98.1/X software.^11^To get the collective picture, multi-dimensional scaling and hierarchical cluster analysis of pile sort data was undertaken. To increase the validity of the results, the findings of the free list, pile sort exercise and FGD were presented to the participants.

Later, the findings were translated and typed out in the local language Marathi and then circulated to 26 KVMs (with 15-20 members each) in surrounding villages for sharing and discussion during their monthly village-based meetings. A social worker facilitated this meeting in all KVMs and obtained feedback from the other group members. These monthly meetings were of one to two hours duration and were held in the evening when most of the members are back from their agricultural work. This activity was carried out over the period of one month.

Based on the pile sorting exercise, a focus group discussion with ten representative KVM members and feedback from all 26 KVM members were summarized. The third column presents the action proposed or being undertaken by the state and central government. Italic text signifies direct quoting from the participants.

## Results

In the free list exercise, the various eleven reasons identified for farmers’ suicide in our area in descending order of Smith’s S value were as follows. 1) debt, 2) addiction, 3) environmental problems, 4) poor prices for farm produce, 5) stress and family responsibilities, 6) government apathy, 7) poor irrigation, 8) increased cost of cultivation, 9) private money lenders, 10) use of chemical fertilizers 11) crop failure. These items were then subjected to pile sorting exercise (Table 1).

**Table:** Table 1:**Reasons for farmers suicides in the Vidarbha region of Maharashtra, India**

Reasons for farmers’ suicide	Frequency across list	Average rank	Smith’s S value
Debt	5	2.2	0.592
Addictions	2	1.5	0.265
Environmental problems	2	1.5	0.257
Poor price for farm produce	2	2.5	0.224
Stress and family responsibilities	2	4.0	0.163
Government apathy	2	3.5	0.159
Poor irrigation	1	1.0	0.143
Increased cost of cultivation	2	3.5	0.143
Private money lenders	2	3.5	0.136
Use of chemical fertilizers	3	5.6	0.129
Crop failure	2	4.0	0.124

As found in the analysis of the pile sort data, eleven perceived reasons for farmers’ suicides were clustered into five groups, which they thought of as mutually related to each other and suggested solutions for each group of problems. The solutions suggested for each group of problems was compiled in the second column of Table 2.

**Table:** Table 2:**The participants then formed groups of related reasons and suggested solutions for these group of problems (obtained from multidimensional clustering)**

Reasons (from pile sort)	Suggested solutions (from free list exercise with descending Smith’s S value)	Actions proposed or taken by the government (Central and State)12
Crop failure, poor irrigation and increased cost of cultivation	1.Improve soil quality (soil testing)	Subsidiary income opportunities through horticulture,
	2.Organic farming	livestock, dairying, fisheries etc.
	3.Irrigation (well, canal, water harvesting)	
	4.Low cost management of farming	
	5.Support business with farming	Assured irrigation facilities.
	6.Crop insurance	Effective watershed management
Government apathy and poor price for farm produce	1.Farmer should sell their farm produce on their own	
	2.Do not rely much on government	
	3.Farmers’ group can process raw products	
	4.Farmers should have their own warehouse	
	5.Farming as per market demand	
Use of chemical fertilizers and environmental problems	1.Organic farming	Seed replacement program.
	2.Promote farm-saved seeds	Organic Farming Technology Mission
	3.Low cost farming	
Stress,family responsibilities	1.Ensure women’s participation	
	2.Camps for stress relief	
	3.Counseling	
	4.Family planning	
Debt, addictions and money lenders	1.Avoid addictions	Ban on illegal private lending.
	2.Avoid loans from private money lenders	Disbursing crop loans through SHGs.
	3.Farmers club formation	Financial assistance for mass community
	4.Hard work on farm	marriages
	5.Avoid expenditure on rituals	

Subsequently, in a focus group discussion, a participant said, “Nowadays there is no respect and dignity for farm workers. The government announced the relief package for the farmers but they are mere passive recipients of it and no efforts are being made in the direction of farmers’ self-reliance for the future”. In order to ensure self-reliance, farmers wanted capacity building and training on newer techniques of farming. We should be taken on study visits to other states or neighboring countries where farmers are working successfully against the adverse environmental conditions.

Even when the government has announced the relief package for farmers, suicides are still going on. In response to this one of the participating farmers suggested the need to develop a ‘support system’ for the farmers and said, “This has become a disease now. Similar to disease conditions such as malaria, filarial etc, there should be a monitoring system to identify vulnerable, poor, small-scale farmers and solve their problems”.

The government encourages farmers to develop alternative sources of income. In response to this, farmers remarked, “the produce of such alternative sources should have market demand. Relief packages in the form of farm equipment are being distributed through the district or taluka level bureaucratic government system. Poor and needy farmers avoid going to district or taluka level as that requires frequent visits. Hence, to make the service accessible and bring transparency, participants said that such distribution should be done at village level ‘Gram-sabha’ [local self-help government] where all the villagers assemble and watch the process. This process will prevent manipulation by the distributing officers and the siphoning off of the poor farmers’ benefit by rich farmers”. Another farmer said, “The benefit from the government package goes to middle level or large scale farmers. Arranging a loan from the bank is a lengthy procedure and banks avoid giving loans to small farmers who have poor capacity to repay the loan. Hence, these poor small farmers go to private money lenders who verbally negotiate the business. He will be repaying such loans until his death.”

## Discussion

In the present study, farmers perceived debt, addiction, environmental problems, poor prices for farm produce, stress and family responsibilities, government apathy, poor irrigation, increased cost of cultivation, private money lenders, use of chemical fertilizers and crop failure as the most significant reasons for farmers’ suicides. Participants suggested solutions to these groups of problems. The major themes that emerged from the FGD were self-reliance and capacity building of farmers, a monitoring and support system for vulnerable farmers and a village-level, transparent system for the disbursement of relief packages.

According to a study conducted by the Indira Gandhi Institute of Development Research, Mumbai, the major reasons for farmers’ suicides are debt, crop failure and low return, illness of family members, failure to arrange marriage of daughters and a lack of alternative sources of income.^12^In our study, participating farmers perceived farmer suicides as a complex interplay of eleven reasons which cover social, political and environmental constraints. Participants perceived that the human activities such as excess use of chemical fertilizers and use of genetically modified seeds cause a loss of land biodiversity and repeated crop failure, which subsequently lead to high costs of cultivation and debt. It was aggravated by government policies related to market prices, exploitation by private money lenders and its ultimate health consequence was frustration leading to suicide.

As found in our study, farmers are losing faith in the government due to its failure to design and implement pro-poor policies for the majority of small farmers who survive on agriculture. Many states have offered financial relief packages only to the families of deceased farmers who were unable to manage payments on their bank loans. Provision of relief facilities alone is not sufficient as it has been observed in the case of Andhra Pradesh where farmers committed suicide to enable their families to partake of the benefits of relief packages.^13^In the present study, farmers have suggested the development of a monitoring system to identify vulnerable farmers and offer them timely help.

In the absence of institutionalized finance, the farmers normally resort to borrowing from private money lenders. Significantly, the loans taken from the private moneylenders are difficult to repay due to high interest rates. Hence, the government should ensure institutional finance and crop insurance to small farmers. Maharashtra state government also plan to alleviate debt, ensure fresh crop loans for both small and big farmers, disburse loans through the farmers’ Self Help Groups, provide subsidy of the crop insurance premium, encourage the promotion of agro-processing industry, provide financial assistance for community marriage and encourage organic farming.^14^ As suggested by study participants, to ensure transparency, its disbursement should be done through village-level Gram-sabha which is crucial to regain the faith of poor small scale farmers and ensure their survival. In the United States, there was a rise in farmers’ suicides after the Great Depression. To counter this, the government started a farmers’ insurance program, which is the only major federally-managed insurance program in existence.^1^

Farmers expressed their concerns for chemical fertilizers and environmental degradation. The nutrients of the soil are being destroyed by the over-use of pesticides and chemical fertilizers needed to successfully grow the genetically modified seeds. This repeated degradation will result in the loss of land productivity thus putting future generations of farmers at even greater risk of poverty and famine.^15^In the present study, participants suggested the promotion of organic farming and reducing dependency on commodities such as chemical fertilizers, pesticides, and genetically modified seeds. In rural China, chronic pesticide exposure was found to be associated with suicidal tendencies, which supports findings from previous studies.^16^Given the high level of pesticide exposure and high suicide risk, a clarification of the causal mechanisms underlying this association and development of appropriate interventions are priorities for public health and health policy.

The need for stress relief camps and counseling services for farmers was expressed. Walker et al reported that even in the absence of psychiatric morbidity, farmers were more likely to report that life is not worth living compared with the general population.^17^In Australia, a strong correlation between droughts and suicide rates among farmers was found. Hence, if a drought was predicted, there was rapid mobilization of social workers, psychologists and psychiatrists to the drought-hit region along with other supportive measures while in India action is predominantly limited to political announcement of exgratia benefits and not towards prevention strategies.^1^In India, there is a need to strengthen the National Mental Health Program at primary health care level so as to offer support and counseling to vulnerable farmers in rural areas.

The present field-based formative study explored the farmers’ perceived causes of suicides and their solutions. These findings may be useful for policy formulation at local level. The limitations of the present study should be kept in mind. It was a small scale study conducted in a limited geographical area. Hence, further research at a wider level is required to confirm our findings. In conclusion, the farmers’ suicides in Vidarbha are due to the complex interplay of social, political and environmental constraints. Hence, a comprehensive intervention to ensure self-reliance and capacity building of farmers in modern farming techniques, a monitoring and support system for vulnerable farmers and a transparent, village-level system for disbursement of relief packages is required to prevent farmers’ suicides in the near future. These suggested interventions are consistent with the recent recommendations by an autonomous administrative training institute by the government of Maharashtra.^5^ Apart from this, there is a need to strengthen the National Mental Health Program at primary health care level to offer support and counseling to vulnerable farmers in rural areas.
